# Inhibition of vascular endothelial growth factor by small interfering RNA upregulates differentiation, maturation and function of dendritic cells

**DOI:** 10.3892/etm.2014.2059

**Published:** 2014-11-11

**Authors:** HAIYAN WANG, LUPING ZHANG, SHAOYAN ZHANG, YANNIAN LI

**Affiliations:** 1 Department of Transfusion, The Affiliated Hospital of Qingdao University, Qingdao University, Qingdao, Shandong 266033, P.R. China; 2 Clinical Laboratory, Qingdao Hiser Hospital, Qingdao University, Qingdao, Shandong 266033, P.R. China; 3 Clinical Laboratory, The Affiliated Hospital of Qingdao University, Qingdao University, Qingdao, Shandong 266033, P.R. China

**Keywords:** dendritic cells, vascular endothelial growth factor, small interfering RNA, peripheral blood monouclear cells, cytotoxic T lymphocyte, MCF-7

## Abstract

This study aimed to investigate the effects of vascular endothelial growth factor (VEGF) secreted by MCF-7 breast cancer cells on the differentiation, maturation and function of dendritic cells (DCs). Small interfering RNAs (siRNAs) directed against the VEGF gene were designed and transfected into MCF-7 breast cancer cells at an optimal concentration (100 nmol/l) using cationic liposome transfection reagent, whereas the control group was transfected with only transfection reagent. Western blot analysis and ELISA were used to determine VEGF protein expression and VEGF concentration, respectively. Mononuclear cells were cultured with the culture supernatants from primary MCF-7 cells (control group) and siRNA-treated MCF-7 cells (siRNA group). The DC phenotypes, including CD1a, CD80, CD83, CD86 and HLA-DR, were evaluated by flow cytometry. The MTT assay was used to assess the cytotoxicity of DC-mediated tumor-specific cytotoxic T lymphocytes (CTLs) against MCF-7 cells in the two different culture supernatants. The VEGF-targeted constructed siRNA inhibited VEGF expression in MCF-7 cells. Cultivation with the culture supernatants from MCF-7 cells treated with siRNA affected DC morphology. DCs in the siRNA group exhibited a significantly higher expression of CD86, CD80, CD83 and HLA-DR compared to the cells in the control group, whereas the expression of CD1a in the siRNA group was significantly lower compared to that in the control group. The cytotoxic activity of CTLs mediated by DCs was significantly altered by siRNA transfection. These results indicated that VEGF may play a significant role in tumor development, progression and immunosuppression.

## Introduction

Dendritic cells (DCs) are potent bone marrow-derived antigen-presenting cells (1). DCs constitute a complex system of cells that is able to induce primary immune responses (2–4). In addition, DCs are effective in stimulating secondary immune responses (5). Thus, these cells play a central role in antitumor immunity. However, the function of the immune system in tumor-bearing hosts is often severely compromised, particularly in hosts with advanced-stage disease, who present with a diminished ability to activate immune responses against the tumor. Tumor cells appear to have developed mechanisms to inhibit immune system recognition and control (6). DCs may represent a target for the inhibition of antitumor immune responses (7).

Defective function of DCs in cancer has been reported by several study groups (8–12); however, the causes of DC impairment have not been fully elucidated. One of the possible mechanisms underlying DC dysfunction in cancer is the abnormal functional maturation of these cells from their progenitors (13,14) caused by tumor-derived factors (15–20). Despite significant advances in the understanding of the mechanisms responsible for cancer cell transformation and proliferation, the immunological pathophysiology of cancer patients, particularly the antitumor cytokine network, requires further investigation.

Angiogenetic processes appear to be regulated by cytokines. The cytokine vascular endothelial growth factor (VEGF) induced by hypoxia is produced by almost all tumors. VEGF directly stimulates the growth of vascular endothelial cells and the formation of tumor neovasculature (21–23). Abnormally high blood concentrations of VEGF have been shown to be associated with poor prognosis in solid as well as hematological malignancies (24). Inhibition of angiogenesis may be one of the mechanism through which the activation of effective anticancer immunity controls neoplastic growth (25–28).

In this study, we investigated the effect of culture supernatants from fresh primary MCF-7 cells on DCs by evaluating the effect of small interfering RNA (siRNA) targeting VEGF, in an attempt to elucidate the association between VEGF and impaired DC differentiation. We cultured mononuclear cells from human peripheral blood mononuclear cells (PBMCs) in the presence of culture supernatants from fresh primary MCF-7 cells to evaluate the association of impaired DCs with tumor-derived factors. Subsequently, we downregulated VEGF expression by siRNA in MCF-7 cells and evaluated the effects of VEGF on DCs.

## Materials and methods

### siRNA design

The siRNA sequences that target human VEGF were constructed according to established guidelines (29). The primer sequences were 5′-GGAGUACCCUGAUGAGAUCUU-3′ (forward) and 5′-GAUCUCAUCAGGGUACUCCUU-3′ (reverse). The negative control scramble siRNA (siRNA_SCR_) primer sequences were 5′-GCGUAACGCGGGAAUUUACUU-3′ (forward) and 5′-GUAAAUUCCCGCGUUACGCUU-3′ (reverse). These sequences were verified by DNA sequencing according to the manufacturer’s instructions (Guangzhou RiboBio Co., Ltd., Guangzhou, Guangdong, China).

### MCF-7 cell culture and transfection

The MCF-7 human breast cancer cell line was kindly provided by the Cell Culture Center of the Peking Union Medical College of China and was cultured in RPMI-1640 medium containing 10% fetal calf serum in a 37°C humidified 5% CO_2_ incubator. The medium was changed every two days. To maintain the cells at optimal proliferating conditions, they were passaged at 80% confluence and seeded at 20% confluence. Transfection was performed at ~90% confluence using Lipofectamine™ 2000 (Invitrogen Co., Carlsbad, CA, USA) following the manufacturer’s protocol. The cells were collected at 48 h following transfection and used for western blot analysis.

### ELISA

The VEGF concentration in the conditioned medium of MCF-7 cells was measured using a commercially available human VEGF ELISA kit (Chemicon, Millipore Corporation, Temecula, CA, USA). MCF-7 cells were plated on a 96-well plate at a density of 6×10^3^ cells/ml. After 24 h of culture, the cells were transfected with siRNA (25 nmol/l, 50 nmol/l, 100 nmol/l and 200 nmol/l), siRNA_SCR_, or Lipofectamine reagent overnight at 37°C. The cultures were then washed twice with Hanks’ Balanced Salt solution and incubated with fresh medium for an additional 48 h. The supernatants were collected and VEGF concentration was quantified (pg/ml) by ELISA according to the manufacturer’s recommendations to determine the effect of specific downregulation and the optimal concentration of siRNA transfection.

### MTT assay

Using a 96-well plate, a total of 8×10^3^ MCF-7 cells were seeded in each well and allowed to attach for 18 h. The cells were later treated with siRNA (25 nmol/l, 50 nmol/l, 100 nmol/l or 200 nmol/l) or siRNA_SCR_. At 48 h following transfection, 20 μl MTT (5 mg/ml) was added to the cells in each well. The cells were subsequently incubated for 4 h, followed by the addition of 100 μl dimethyl sulfoxide and the cells were incubated for another 15 min. The optical density was determined at 492 nm with a microculture plate reader (SpectraMax M2; Molecular Devices, Sunnyvale, CA, USA). To determine the inhibition rate, the absorbance values were normalized to the values obtained from the blank control group of cells.

### Western blot analysis

Following treatment with 100 nmol/l siRNA or siRNA_SCR_ for 48 h, the MCF-7 cells attached to culture dishes were trypsinized and washed in cold phosphate-buffered saline (PBS). Briefly, 100 μg protein samples were subjected to 10% standard SDS-PAGE, with prestained molecular weight markers being run in parallel to identify VEGF protein. Subsequently, the resolved proteins were transferred to polyvinylidene difluoride membranes. The membranes were then incubated with mouse monoclonal anti-VEGF antibody (cat. no. sc-7269; Santa Cruz Biotechnology, Inc., Santa Cruz, CA, USA) and mouse monoclonal anti-β-actin antibody (cat. no. sc-47778; Santa Cruz Biotechnology, Inc.). antibodies. Following extensive washing, the membranes were incubated with anti-mouse IgG-horseradish peroxidase-conjugated antibody for 1 h at room temperature and developed with a Luminol chemiluminescence detection kit (Promega Corporation, Madison, WI, USA). Protein expression was quantified with a Gel EDAS analysis system and Gel-Pro Analyzer 3.1 software (Shenzhen Tianneng Corporation, Shenzhen, Guangdong, China).

### DC culture

Human PBMCs were obtained from heparinized blood of healthy volunteers by density gradient centrifugation following informed consent. The cells were cultured for 5–6 days in complete RPMI-1640 medium [GlutaMAX (Invitrogen Co.), supplemented with 10% fetal calf serum, 100 U/ml penicillin and 100 g/ml streptomycin] with 100 ng/ml granulocyte-macrophage colony-stimulating factor (GM-CSF) and 20 ng/ml interleukin-4 (IL-4). Maturation was induced for 48 h in the presence of 10 ng/ml tumor necrosis factor-α (TNF-α). The DCs were divided into the siRNA and control groups. The DCs of the siRNA group were cultured in RPMI-1640 medium with the addition of the culture supernatant of MCF-7 cells transfected with siRNA (optimal concentration of 100 nmol/l) according to the volume ratio of 1:2 (supernatant vs. RPMI-1640). The DCs of the control group were cultured in RPMI-1640 medium with the addition of the culture supernatant of MCF-7 cells transfected with siRNA_SCR_ (100 nmol/l) according to the volume ratio of 1:2 (supernatant vs. RPMI-1640).

### DC morphology imaging and flow cytometry

The DCs in the two groups were observed daily under an inverted Olympus microscope (Olympus Optical Co., Ltd., Tokyo, Japan) with a green light filter. Following 8 days of culture, the DCs were stained by standard direct procedure using phycoerythrin-conjugated mouse monoclonal antibodies (mAbs) against CD1a (cat. no. 555807), CD80 (cat. no. 557227), CD83 (cat. no. 556855), CD86 (cat. no. 555658) or HLA-DR (cat. no. 555812; all from BD Biosciences, Franklin Lakes, NJ, USA). The cells were incubated with mAbs for 20–30 min at ambient temperature, washed twice with PBS and resuspended in 100 μl PBS. Cell fluorescence was analyzed by the Epics XL flow cytometer (Beckman Coulter Inc., Brea, CA, USA).

### Mixed lymphocyte reaction

Responder T cells were purified from the PBMCs of healthy volunteers. T cells (1×10^6^/ml) were co-cultured with DCs loaded with MCF-7 antigen for 72 h to induce cytotoxic T lymphocytes (CTLs). The CTLs were then collected and used as the effector cells in CTL assays. As the target cells, MCF-7 cells were placed in 96-well culture plates at lx10^4^ cells per well and co-cultured with the effector cells at varied effector/target cell ratios (E/T) of l:10, 1:25 and l:50 for 48 h. The cytotoxic activity was detected with the MTT assay. The tests were performed in triplicate and the results are expressed as mean counts per minute with standard deviation.

### Statistical analysis

Statistical analyses were performed using SPSS software, version 11.0 (SPSS Inc., Chicago, IL, USA). Data are expressed as the means ± SD. The results were considered statistically significant if P<0.05 was obtained by the appropriate ANOVA procedure and the Student’s t-test.

## Results

### VEGF-targeted siRNA inhibits the expression of VEGF in MCF-7 cells

**Figure 1:**
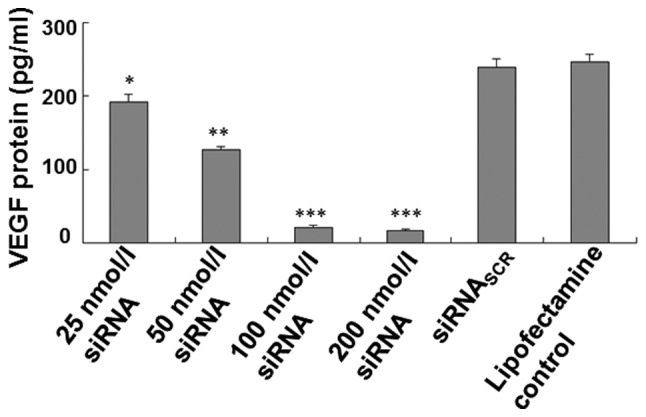
Vascular endothelial growth factor (VEGF) protein levels in response to different small interfering (si)RNA concentrations. The histograms represent VEGF protein levels in the presence of siRNA (25, 50, 100 and 200 nmol/l), scramble siRNA (siRNA_SCR_) and Lipofectamine control. Data are presented as means + standard deviation (n=3). ^*^, values significantly different from siRNA_SCR_ (P<0.05); ^**^, values significantly different from 25 nmol/l siRNA (P<0.05); ^***^, values significantly different from 50 nmol/l siRNA (P<0.05).

**Figure 2:**
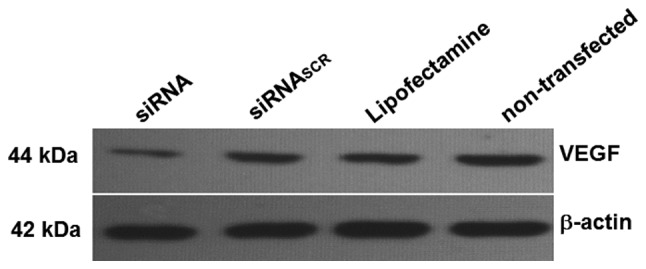
Vascular endothelial growth factor (VEGF) protein expression in MCF-7 cells as detected by western blot analysis. The small interfering (si)RNA directed against the VEGF gene was transfected into MCF-7 cells at the optimal concentration (100 nmol/l) using Lipofectamine 2000, whereas the control group was transfected with only Lipofectamine 2000. β-actin was used as an internal control. siRNA_SCR_, scramble siRNA.

In order to test whether siRNAs affect the expression of VEGF, we measured the VEGF concentration in the culture supernatants of MCF-7 cells transfected with siRNA (25 nmol/l, 50 nmol/l, 100 nmol/l or 200 nmol/l), siRNA_SCR_ or Lipofectamine reagent using ELISA and analyzed the proteins by western blotting. The ELISA data demonstrated that the VEGF expression and protein production in the supernatants of MCF-7 cells transfected with siRNA (100 nmol/l and 200 nmol/l) were significantly decreased compared to that in the controls; however, the level of VEGF protein was not significantly different between the 100 and the 200 nmol/l siRNA groups, or among the siRNA_SCR_, Lipofectamine control and the blank control groups (Fig. 1). The western blot analysis demonstrated that VEGF expression was significantly reduced in cells transfected with siRNA compared to those transfected with siRNA_SCR_ or Lipofectamine (P<0.05) (Fig. 1). There was no significant difference among the siRNA_SCR_, Lipofectamine and non-transfected groups (Fig. 2). These data suggested that the constructed VEGF-targeted siRNA inhibited the expression of VEGF.

### Cultivation with the culture supernatants from MCF-7 cells treated with siRNA affects DC morphology

**Figure 3:**
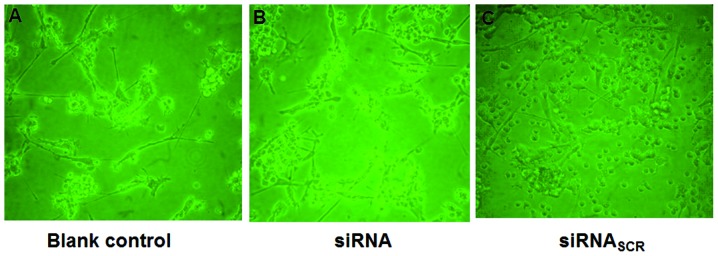
Morphological observation of peripheral blood mononuclear cells in the (A) blank control, (B) small interfering (si)RNA and (C) scramble siRNA (siRNA_SCR_) groups. The microscopic images were captured with a fluorescence microscope on the 8th day.

Optical microscopy was employed to investigate the effect of siRNA on DC morphology. Representative photographs taken on day 8 demonstrated that DCs generated from adherent human PBMCs of healthy donors in the presence of GM-CSF, IL-4 and TNF-α exhibited significant differences in morphology between the siRNA and siRNA_SCR_ groups (Fig. 3). The DCs in the blank control group exhibited typical arborizations (Fig. 3A). The PBMCs cultured with the culture supernatants from MCF-7 cells treated with siRNA started to elongate into irregular shapes on the 2nd or 3rd day and exhibited the morphological characteristics of DCs on the 8th day (Fig. 3B). The DCs in the siRNA_SCR_ group, cultured with the culture supernatants from MCF-7 cells transfected with siRNA_SCR_, started to elongate marginally on the 3rd or 4th day, but exhibited no distinct DC characteristics until the 8th day (Fig. 3C). These data demonstrated that DC morphology was affected by cultivation with the culture supernatants from MCF-7 cells treated with siRNA.

### siRNA alters the effect of VEGF on DC surface phenotypes

In order to investigate the effects of VEGF on DC differentiation in the culture supernatants from MCF-7 cells and determine how this effect is affected by VEGF-targeted siRNA, DCs were labeled with a mixture of phycoerythrin-conjugated lineage-specific antibodies (anti-HLA-DR, CD80, CD83, CD1a, CD86) and analyzed directly using flow cytometry. The expression of HLA-DR on DCs in the siRNA group was higher compared to that in the control group that was transfected with siRNA_SCR_ (Table I). Folllowing siRNA transfection, the percentage of cells expressing CD80, CD86 and CD83 was increased (Table I). However, the expression of CD1a in the siRNA group was lower compared to that in the control group (Table I). These data demonstrated that siRNA altered the effect of VEGF on DC surface phenotypes.

### siRNA enhances the toxicity of CTLs induced by DCs

In order to detect the inhibition rates of tumor-specific CTLs against MCF-7 cells mediated by DCs cultured in different media, the MTT assay was used. The results of the MTT assay demonstrated that the inducing activity of DCs in the siRNA group was enhanced in comparison to DCs in the control group (siRNA_SCR_). The cytotoxic activity of CTLs was most significant at E/T 1:50 (P<0.01) (Table II). These data indicated that the cytotoxic activity of CTLs mediated by DCs was significantly increased by transfection with siRNA.

## Discussion

DCs play a central role in the induction of antitumor immune responses (30,31). Adequate DC function is crucial for effective antitumor control and successful cancer immunotherapy. The inadequate function of DCs in cancer may be one of the important mechanisms through which tumors escape immune system control. Cancer patients, particularly those with advanced-stage disease, have diminished ability to activate immune responses against the tumor. Although the mechanisms underlying this immune ‘defect’ are multifactorial, DC dysfunction plays a key role (32–36). DCs not only initiate T-cell responses, but are also involved in silencing T-cell immune responses. The functional activities of DCs mainly depend on their state of activation and differentiation. Terminally differentiated mature DCs may efficiently induce the development of effector T cells, whereas DCs are also involved in the maintenance of peripheral tolerance. However, accumulated DCs that are educated at the tumor site, act as functional inhibitors of tumor-specific immune responses in cancer. Our study demonstrated that the culture supernatants from primary MCF-7 cells inhibited the differentiation, maturation and function of DCs induced from the PBMCs of healthy donors.

We demonstrated that DCs cultured in the culture supernatants from MCF-7 cells transfected with VEGF-targeted siRNA exhibited upregulated expression of CD80, CD83, CD86 and HLA-DR, but a lower level of CD1a expression. T-lymphocyte stimulating activities and the capacity to induce CTL cytotoxicity were all enhanced. Our results strongly suggest that the soluble factor VEGF secreted by MCF-7 cells played an important role in tumor evasion from immune surveillance, which is one of the factors responsible for defective DC maturation (37). However, the detailed mechanisms underlying the effect of VEGF on DC maturation and function have not been fully elucidated. It was previously indicated that DCs play a dual role in immune regulation through cross-priming of T cells and immunosuppression (38). Dikov *et al* (39) reported that the direct impairment of DC function by VEGF is mediated primarily by VEGFR1/Flt-1. Therefore, further studies are required to determine the detailed mechanisms of the effect of VEGF on human DCs.

VEGF is produced by almost all tumor cells and is responsible for the formation of tumor neovasculature (40). Our data indicated that the designed siRNAs specifically inhibited VEGF expression in MCF-7 cells and the inhibition of DC maturation and function in the culture supernatants from siRNA-transfected cells was significantly decreased. These data suggested that VEGF may play a significant role in tumor development, progression and immunosuppression. VEGF-targeted siRNA may be effective in the induction of antitumor immune responses and the treatment of breast cancer.

## Figures and Tables

**Table I:** Expression of dendritic cell phenotypes cultured during 8 days.

Group	CD1a	CD80	CD83	CD86	HLA-DR
siRNA	14.56±1.26^a^	81.45±2.27^a^	82.24±3.24^a^	91.15±2.71^a^	98.64±2.35^a^
Control	48.26±2.26	34.21±2.11	48.34±2.74	36.82±1.67	58.12±2.12

Data are presented as means ± standard deviation (n=3).

a Values significantly different from the control group (P<0.01).

siRNA, small interfering RNA.

**Table II:** Killing abilities of CTLs in different groups.

Group	E/T=1:10	E/T=1:25	E/T=1:50
siRNA	26.1±4.6^a^	36.2±3.6^a^	66.3±6.8^a^
Control	4.9±2.0	10.4±2.1	17.3±1.8
T cells	3.1±1.3	3.5±0.8	3.04±1.7

Data are presented as means of percentage ± standard deviation (n=3)

a Values significantly different from the control group (P<0.01).

CTLs, cytotoxic T cells; siRNA, small interfering RNA; E/T, effector/target cell ratio.
